# Biofeedback Based Home Balance Training can Improve Balance but Not Gait in People with Multiple Sclerosis

**DOI:** 10.1155/2019/2854130

**Published:** 2019-12-23

**Authors:** Klara Novotna, Marketa Janatova, Karel Hana, Olga Svestkova, Jana Preiningerova Lizrova, Eva Kubala Havrdova

**Affiliations:** ^1^Department of Neurology and Centre of Clinical Neuroscience, First Faculty of Medicine, Charles University and General University Hospital in Prague, Prague, Czech Republic; ^2^MS Rehab z.s., Prague, Czech Republic; ^3^Department of Rehabilitation Medicine, First Faculty of Medicine, Charles University and General University Hospital in Prague, Prague, Czech Republic; ^4^Joint Department of Biomedical Engineering CTU and Charles University, Prague, Czech Republic

## Abstract

**Background:**

Impaired balance is common in people with multiple sclerosis (MS) and can be present even in those with a mild disability level. With increasing disability, gait, and balance impairment progress, and lead to increased risk of falls. In some recent studies, interactive commercial video games were used for improving balance, but their limitation is their lack of individual training parameter settings needed for rehabilitation purposes. The aim of this study was to evaluate the feasibility and effect of balance exercise in the home setting using the rehabilitation Homebalance® system.

**Methods:**

A single-centre, controlled, single blind study with allocation to intervention group or to control group was utilised. Participants were assessed at baseline, after four weeks of home-based balance training, and follow-up after four weeks. The primary outcomes were the Berg Balance Test (BBT). The secondary outcome measures included the Mini-BESTest, Timed Up, and Go Test (part of Mini-BESTest), and spatio-temporal gait parameter evaluation using the GAITRite instrument. The patient reported outcomes (PRO) included the 12-Item MS Walking Scale, Activities-specific Balance Confidence Scale, and the Falls Efficacy Scale.

**Results:**

A total of 39 people with Multiple Sclerosis (10 men) were enrolled into the study. The mean age of participants was 40.69 ± 10.2 years, with a mean disease duration 14.76 ± 9.1 years and mean disability level 3.8 ± 1.9 EDSS (EDSS range 1.5–7). Statistically significant improvements within the home exercise group were present for the BBT and the Mini-BESTest. This improvement was more significant in the subgroup with moderate and higher disability (EDSS 4.5–7). All other gait parameters and PRO did not show any improvement. Follow-up assessment after four weeks showed that the reached improvement persisted for a short time period after finishing the regular training regimen.

**Conclusion:**

In comparison with no intervention, a short-term programme of home-based balance training using Homebalance® improved balance but not gait performance in a group of people with MS. It seems that home-based balance training tailored according to individual needs by a physiotherapist may be a future approach to consider for telerehabilitation of people with MS.

## 1. Background

Multiple sclerosis (MS) is a chronic neurologic condition with many symptoms, which can have a negative influence on balance (impaired sensitivity, muscle weakness, spasticity, movement incoordination, cognitive dysfunction, slowed somatosensory conduction, and impaired central integration) [1].

Balance impairment is a common finding in people with MS and can be present even in those with a mild neurological disability [2–4]. This impairment is characterised by increased sway in quiet stance, delayed anticipatory, and automatic postural adjustment, and reduced ability to move towards the limit of stability [5]. Poor balance performance on static and dynamic balance tests is associated with an increased number of falls [6]. All this often prevents people from performing their daily living activities [7].

In the past decade much attention was directed towards balance rehabilitation in patients with MS. Balance rehabilitation appeared to be a useful tool in reducing the fall rate and improving balance performance in subjects with MS. One of the goals of balance rehabilitation is to reduce the number of falls; however, the frequency of falls cannot be considered the only outcome for patients with MS. A recent systematic review concluded that physiotherapy has a small but significant beneficial effect on balance in people with MS with mild and moderate disability [8].

Common therapeutic strategies to promote improvements in balance include individually tailored balance programmes, vestibular rehabilitation programmes, resistance, and aerobic training, gait training, and interactive videogames (using commercial balance games or more sophisticated virtual reality programmes) [9].

Individually tailored programmes supervised by a physiotherapist covering both motor and sensory strategies were found to improve balance performance and reduce the number of falls more efficiently than motor strategies alone [10, 11]. Another therapeutic strategy is exercise focused on core stability exercise [12–14].

Other possible approaches may be to use interactive videogames, which are becoming increasingly popular in different patient groups including those with MS [15]. Several studies have shown possible benefits of this type of training using Nintendo Wii either led by a physiotherapist [16–20] or in-home settings [21–23]. Unfortunately, an individual exercise setting in these commercial interactive videogames is limited and production of both types of these commercial games has ceased. Therefore, a new exercise tool, Homebalance®, that enables individual exercise parameter settings, was developed.

The aim was to evaluate the effects of four weeks of balance training in a home setting in a sample of people with MS with a new exercise tool, Homebalance®, providing audio-visual biofeedback. The hypothesis was that, after completion of the programme, balance, and gait performance would have improved and be significantly better in comparison with no exercise.

## 2. Methods

### 2.1. Participants

Patients with MS were selected from outpatients sent for rehabilitation treatment. All patients are followed at the MS centre in the Department of Neurology and Centre of Clinical Neuroscience, First Faculty of Medicine, Charles University and General University Hospital in Prague. All subjects had their MS defined following the McDonald criteria, and had reported subjective perceived imbalance or history of falls (in the last year). In this study, we included subjects: (i) who were clinically stable, without relapse or worsening in the previous three months; (ii) aged 18–60 years with; (iii) ability to walk with or without a walking aid for at least 5 m (EDSS 1–7); and (iv) ability to maintain a standing position for at least 10 minutes, to be able to perform exercise (assessed by physiotherapist).

The exclusion criteria included: (i) inpatient rehabilitation programme during the previous three months; (ii) orthopaedic problems or other conditions affecting balance and gait performance; (iii) blurred vision; (iv) severe cognitive impairment or psychiatric disorders; (v) pregnancy; (vi) weight over 150 kg (to be able to use the exercise platform). Additionally, those participants were not receiving other physiotherapy targeting balance problems or were not having any other changes in lifestyle prior to or during the study.

Recruitment took place between January 2016 and March 2017, with the last follow-up conducted in May 2017. All participants provided written informed consent before entering the study. This trial was approved by the Ethical Committee of the First Faculty of Medicine and General University Hospital in Prague, Czech Republic. The registration number of the study is ISRCTN11744221.

### 2.2. Intervention

**Figure 1:**
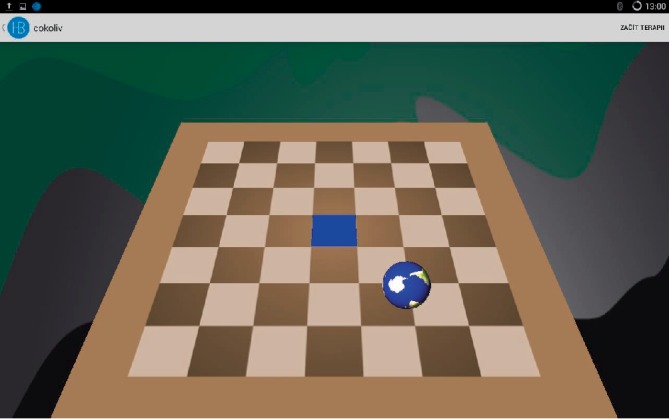
Therapeutic game-Chessboard.

**Figure 2:**
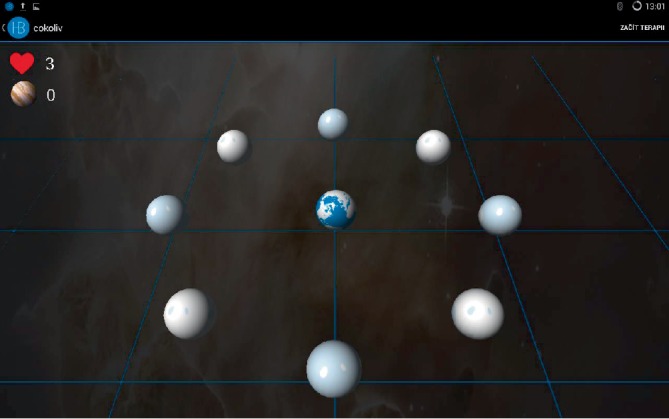
Therapeutic game-planets (dual task-balance and cognitive).

The design of the study was a single-centre wait list randomised controlled study (using a baseline control period). The intervention consisted of individually tailored home-based balance exercise training using Homebalance®. The instructions for participants was to perform the balance exercise at least 15 minutes every day for four weeks (approx. 7 hours of individual tailored balance training). There were two therapeutic games available: (a) a chessboard—where the therapeutic task can be set to different positions/directions; (b) planets—where the therapeutic task is to increase the limits of stability combined with cognitive training (remember the order of the planets) (Figures 1 and 2). Participants were instructed to play both games. During the first therapeutic session, a physiotherapist instructed participants how to perform the exercises and how to maintain a correct upright position. Patients with higher EDSS level were instructed how to perform balance exercise in a safe way (standing in front of table or walker to be able to hold when needed). The participants could sit down and have a rest during the exercise any time they needed. The intervention was tailored individually to suit each participant's balance impairment and needs. As a control group, patients from the waiting list were used. The control group received no intervention.

#### 2.2.1. Therapeutic Tool

**Figure 3:**
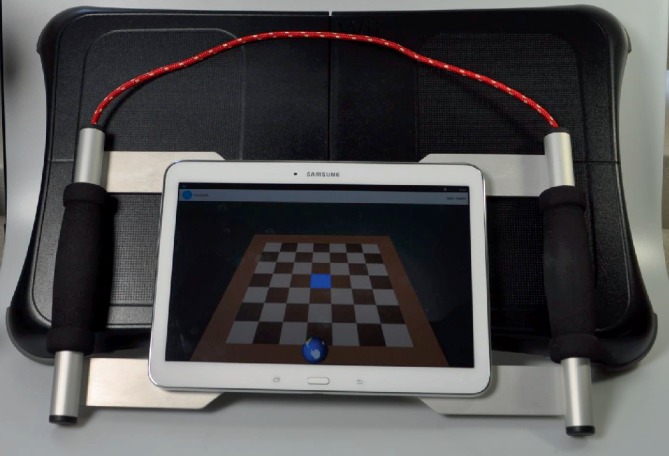
Homebalance system®.

Homebalance® (Clevertech, CZ) is an interactive system for home-based therapy of balance disorders. As the system is intended to be used in home-based rehabilitation, it consists of low-cost, portable, and lightweight components (Figure 3). These include: tablet computer (size 10.1”) with diagnostic-therapeutic software (developed by the Spin-off company and research results commercialization center at the 1st Faculty of Medicine, Charles University in Prague) and a portable stabilometric platform (Wii balance platform board, 53 × 32 × 5 cm, maximum load 150 kg). The sensitivity of platform for postural sway of participant can be tailored according to individual patient needs (for example make it more sensitive for spastic patients or less sensitive for people with ataxia). It is also possible to set a preferable direction and range of movement of patients postural sways in the therapeutic game. The therapy includes active repetitive games-like training (Figures 2 and 3). Standing on the stabilometric platform, the patient is instructed to move the item (an avatar in therapeutic game) by shifting their centre of gravity. Participants were encouraged to gradually increase the difficulty of the exercise (with prolongation of persistence in position or with decreasing sensitivity of exercise platform). Total exercise time was reported in exercise dairy.

### 2.3. Outcomes

The outcome measures were chosen to cover both self-reported and more objectively measured changes. Outcome measures were assessed at baseline and at four weeks after intervention and at four weeks follow-up. Physical assessments were undertaken in the hospital setting and administered by the study researcher (physiotherapist with clinical experience in neurorehabilitation). The primary outcomes of study were the Berg Balance Test. The secondary outcomes included the Mini-BESTest, the Timed Up, and Go test, and assessment of the spatio-temporal gait parameters.

The patient reported outcome measures using self-reported questionnaires were completed after physical assessment. The protocol consisted of the Falls Efficacy Scale, Activities-specific Balance Confidence Scale, and the 12-Item MS Walking Scale.

#### 2.3.1. Berg Balance Test (BBT)

This scale rates performance from 0 (cannot perform) to 4 (normal performance) on 14 items with a maximum total score of 56. A score of 41–56 indicates a low fall risk, 21–40 a medium fall risk, and 0–20 a high fall risk. The validity and reliability of the scale have been tested on subjects with MS [24, 25].

#### 2.3.2. Mini-BESTest

The Mini-BESTest is a shorter version of the Balance Evaluation System Test (BEST), originally with 36 items, but the shorter version has 14 items with a maximum score of 28 points. This test includes four subscales: anticipatory postural control; reactive postural control; sensory orientation; and stability of gait [26]. One item of the Mini-BEST test is Timed Up and Go test (TUG) that can be used a single balance test too [27].

#### 2.3.3. Gait Evaluation

The gait parameters were obtained using the GAITRite walkway system to measure temporal and spatial parameters: velocity, cadence, step time, and step length during normal and fast walk [28].

#### 2.3.4. Activities-Specific Balance Confidence (ABC) Scale

This is a scale in which the subject rates his or her perceived level of confidence while performing 16 daily living activities. The scores range from 0 to 100, where 100 indicates a high level of confidence in balance skills [29].

#### 2.3.5. Falls Efficacy Scale-I (FESI-I)

This patient self-reported questionnaire assesses the level of concern relating to falls during 16 activities of daily living, ranging from basic to more demanding activities. The score ranges from 16 to 64, the higher the score, the greater the fear of falling [30].

#### 2.3.6. Multiple Sclerosis Walking Scale-12 (MSWS-12)

This self-administrated scale is rating MS-related walking limitations during the previous two weeks. In 12 questions, subjects answer from 1 (not at all limited) to 5 (extremely limited) [31].

### 2.4. Statistical Analysis

Non-parametric statistical hypothesis test were used due to not normally distributed observed values (evaluated by Shapiro-Wilk test). The Wilcoxon signed-rank test was run for the comparison between baseline parameters and parameters after 4 weeks of regular training. The Mann-Whitney test was employed to compare results between two subgroups of patients (with mild versus severe neurological impairment). For the evaluation of the persisting effect after 4 week (follow-up) the non-parametric Friedman test (for 3 dependent samples) and Dunn test were used. The significance level was set at *p* ≤ 0.05.

## 3. Results

The study sample consisted of 39 participants. The mean age of participants was 40.69 ± 10.2 years, with a mean disease duration 14.76 ± 9.1 years and mean disability level 3.8 ± 1.9 EDSS (EDSS range 1.5–7). Eleven subjects used a walking aid in their daily activities. The baseline demographic and clinical characteristics of the exercise and control groups are shown in Table 1. There was no statistical difference in all assessed gait and balance parameters at baseline in the experimental and control groups (evaluated by the Mann–Whitney test).

Overall compliance with the balance exercise was good. There were no dropouts or adverse event during the treatment period. Participants report the total daily exercise time into exercise dairy. The mean length of total balance training exercise duration was 12 minutes (SD 2.3, range 8–25 min). No adverse events were reported. Some participants reported occasional problems with wireless connection of the tablet with the stabilometric platform. The participants expressed that they felt that they became successively better at performing the games.

There was a statistical improvement in the mean BBT and in the Mini-BESTest after completing this home-based balance exercise programme. The other parameters did not reach statistically significant improvement. Patient reported outcome measures did not show any significant improvement either. The results of all assessments are described in Tables 2 and 3. A significant improvement in balance assessment was also present at follow-up. The results from the follow-up assessment in the experimental group are presented in Table 4.

To further explore our data, in a sub-analysis we analysed the effect of exercise on two subgroups of participants: in a group of patients with mild and moderate disability (EDSS 1.5–4) and in a group of people with moderate and severe disability (EDSS 4.5–7). We found a statistically significant difference between these two groups only in BBT. In a subgroup of people with moderate and severe disability (EDSS 4.5–7), the difference before and after four weeks reached statistical significance. Differences in all parameters in these two subgroups are shown in Table 3.

## 4. Discussion

In the present controlled study, we investigated the effect of home-based individual tailored balance rehabilitation in people with MS. The findings from this study demonstrate the feasibility of this type of home-based balance training using new rehabilitation tool Homebalance®.

We have shown that in a group of people with MS, who subjectively perceived balance impairment and performed four weeks of home-based training with the Homebalance® instrument, improvement in mean BBT, and Mini-BESTest can be reached. These results are similar to findings from studies using the commercial videogame Nintendo Wii Balance Board® to improve the mean Berg Balance Test score in a group of patients with MS after 10–12 hours of supervised training [17, 19].

All participants showed very good compliance with this therapeutic video-gaming. A possible explanation is that these audio-visual biofeedback based therapeutic games could be more motivating for patients than standard balance exercises (with no feedback).

After completion of the home-based balance exercise programme, there were statistically significant differences between the exercise and control groups in balance performance (functional balance assessment, BBT, Mini-BESTest) but not in secondary outcomes (TUG and gait parameters).

When comparing the difference at baseline and after training in the BBT in the group of patients with moderate and severe disability (EDSS 4.5–7), a higher significant improvement in patients with severe disability compared to patients with a mild to moderate disability (EDSS 1.5–4) can be reached. That finding is probably based on the fact that in this more disabled subgroup static balance performance is more deteriorated. For these more disabled group of people with MS, with high risk of falling, the balance training is therefore of great clinical importance. As from a recent study we know that in people with MS, the fall risk peak is at EDSS 4 to EDSS 6 [32].

In our total cohort, the BBT showed a mean difference between pre and post scores of 1.9 in experimental group and 0.6 points for controls. Mean change in BBT after training in mild subgroup was 1.1 and in more disabled group was 3.1. The total change in BBT in the experimental group was lower than the minimal clinically important difference, which is considered as three points [33]. However, clinically significant improvement was achieved in the group with greater disability. The lack of clinical improvement may be explained by the fact that the exercise regimen lasted only four weeks with an approximate total duration of seven hours. This is probably too short an exercise duration to reach clinically or subjectively perceived improvements in patient outcome measures, especially for people with progressive MS or severe disability with limited capacity for motor learning, and neuroplasticity [34]. There is still a lack of evidence on how often and how long balance exercise training should be prescribed in order to achieve a clinically meaningful change in people with MS. The evidence for balance training in the elderly suggests that a higher total dose of exercise (≥50 hours of balance training) is needed [35]. Another possible explanation is that home based balance training using Homebalance® instrument is based on static balance exercise (the task in therapeutic games was based on shifting center of pressure in standing position). But real life situations require also proactive and reactive balance capability and these tasks have not been practised during our interventions.

Apart from the BBT, participants in the experimental group reached statistical improvement in the Mini-BESTest, that could be in some patients with subtle balance deficit more sensitive due to assessment of dynamic gait conditions and standing on uneven surface [36, 37].

These results are in contrast with those from the study by Nilsagard et al. in which the authors showed no statistically significant difference in the balance test after a programme of supervised balance exercises using a Nintendo Wii [16]. This lack of significant results could be caused by lower training frequency (participants in these trials performed balance training twice a week only) and shorter total length of exercise (6 hours in total within 6 weeks).

The lack of improvement in PRO may be explained by the fact that the exercise regimen lasted only four weeks with an approximate total duration of seven hours. This is probably too short an exercise duration to reach clinically or subjectively perceived improvements in patient outcome measures, especially for people with progressive MS or severe disability with limited capacity for motor learning and neuroplasticity [34]. There is still a lack of evidence on how often and how long balance exercise training should be prescribed in order to achieve a clinically meaningful change in people with MS. The evidence for balance training in the elderly suggests that a higher total dose of exercise (≥50 hours of balance training) is needed [35].

Functional assessment (TUG test) and gait parameters did not show statistical significant improvement. Actually these results are not very suprising taking into account that in this type of balance training is in static standing position only. This lack of improvement in gait parameters are in contrast to the findings of Prosperini et al. who reported a reduced number of falls after visuo-proprioceptive training using a stabilometric platform and visual biofeedback on a computer screen [20] or home-based training using a Nintendo Wii [21]. Our contrasting results could be due to methodological differences: in particular, they used longer therapeutic sessions (12 sessions lasting 45 minutes supervised by a physiotherapist) or longer duration of home-based training (5 days per week, 30 minutes each day for 12 weeks) versus a minimum of 15 minutes for 4 weeks in our study. The low number of sessions in our study might be an important issue as there is evidence of a positive correlation between the outcome of balance rehabilitation and number of treatment sessions.

The findings of Prosperini et al. also suggest some positive transfer effect of balance training using video games for improvement of cognition in the PASAT test [38].

To date, this is the first controlled trial examining the effect of home-based balance training using the Homebalance system® in the MS population. In accordance with our findings, we are confident that this system is safe and feasible for use in balance rehabilitation for people with MS with mild to severe disability. Moreover, commercial exergaming devices do not allow individual difficulty adjustment that in some cases is necessary to meet physical abilities and treatment goals. In contrast, in Homebalance system® training difficulties and parameters of both therapeutic games could be settled individually. A limitation of the Nintendo Wii balance games is that most balance games require movement in a medio-lateral direction only. Considering that the Nintendo Wii is designed for healthy people for recreational purposes there is a limitation in setting of exercise difficulty and lack of a clear scoring system. It is difficult for a therapist to administer and customise the training according to the individual patient's needs [23]. The other limitation is that feedback provided by a commercial game is not very supportive and could remind participants of their impairment [39]. The Homebalance® system enables individual setting of training parameters (the direction and amplitude of movement, the sensitivity of the stabilometric platform) and simplicity of operation.

The playing commercially available games with Nintendo Wii can be also related with some injuries even in healthy population [40]. Beside that fact we have to take in account that commercially available Nintendo Wii games are no longer produced.

Balance training using Homebalance® seems to be a possible and safe therapeutic option for people with MS and impaired balance. A recent study showed that high-intensity, task-oriented balance training using visual biofeedback from a commercial video game induces new cerebellar connections in patients with MS [41].

The strength of this study is the sub-analysis comparing the effect of this home exercise training in two subgroups of people with MS (with EDSS 1.5–4 and with EDSS 4.5–6.5).

The limitations of this study include first, the short length of therapy (4 weeks only), second, the small number of participants in the experimental and control groups. Third, the number of falls was not part of outcome measurement in this study, so we do not have enough information about transfer of balance improvement into daily life activities.

## 5. Conclusion

Balance training using Homebalance® system with audio-visual biofeedback seems to be a feasible therapeutic option for people with MS with mild to severe disability. Our results show a promising positive effect of this type of balance training, especially in people with moderate to severe disability, but further studies are required to evaluate the effect of longer training and possible influence on falls and cognition.

## Figures and Tables

**Table 1:** Demographic characteristic of participants.

Parameter	Experimental group *N* = 23 mean (SD)	Control group *N* = 16 mean (SD)
Age (years) mean (SD)	39.39 (9.68)	42.56 (10.63)
Gender mean (SD)	6 males	4 males
EDSS mean (SD)	3.93 (1.91)	3.62 (1.89)
EDSS median (range)	4 (1.5–7)	3.75 (1.5–7)
Disease duration (years) mean (SD)	14.95 (8.59)	14.5 (9.88)

**Table 2:** Results of all parameters.

Parameter	Experimental group (*n* = 23)-baseline mean (SD)	Experimental group (*n* = 23)-4 weeks mean (SD)	Wilcoxon *p*	Control group (*n* = 16)-baseline mean (SD)	Control group (*n* = 16)-4 weeks mean (SD)	Wilcoxon *p*
Berg balance scale (0–56)	48.83 (9.44)	50.7 (8.69)	0.001	51.5 (6.16)	52.19 (5.07)	0.189
Mini-BESTest (0–28)	22.39 (5.96)	23.52 (5.99)	0.001	23.13 (5.17)	23.94 (3.8)	0.133
TUG (sec)	12.12 (11.52)	11.5 (9.87)	0.988	9.62 (5.78)	9.17 (5.06)	0.139
TUG-with dual cognitive task (sec)	13.97 (12.96)	12.31 (9.17)	0.128	11.2 (6.92)	10.04 (4.78)	0.132
ABC	70.83 (22.41)	65.99 (27.7)	0.144	70.24 (23.96)	72.7 (26.07)	0.944
FESI-I	29.35 (11.2)	30.30 (13.13)	0.275	25.56 (9.79)	28.94 (12.13)	0.345
MSWS-12	32.52 (15.62)	30.91 (17.4)	0.073	30.5 (15.28)	33.94 (14.59)	0.123
Velocity-normal (cm/sec)	108.96 (42.29)	114.37 (45.98)	0.128	110.47 (35.29)	116.38 (34.41)	0.049
Cadence-normal (number/min)	102.41 (26.97)	105.45 (29.79)	0.001	102.55 (18.31)	104.3 (18.15)	0.001
Step time *L*-normal (sec)	0.66 (0.30)	0.64 (0.30)	0.006	0.62 (0.21)	0.60 (0.16)	0.060
Step time *P*-normal (sec)	0.66 (0.33)	0.68 (0.46)	0.354	0.60 (0.15)	0.59 (0.15)	0.101
Step length *L*-normal (cm)	61.21 (13.88)	64.66 (15.49)	0.140	62.85 (12.84)	65.58 (11.86)	0.011
Step length *P*-normal (cm)	60.72 (13.46)	63.47 (15.81)	0.394	63.32 (13.6)	65.58 (11.86)	0.030
Single leg stance *L*-normal (%)	31.83 (7.05)	32.11 (7.05)	0.009	33.04 (4.3)	33.91 (2.95)	0.098
Single leg stance *P*-normal (%)	32.77 (6.4)	34.27 (7.34)	0.235	32.6 (4.67)	34.29 (3.57)	0.005
Velocity-fast (cm/sec)	144.33 (58.5)	144.08 (58.33)	0.858	146.53 (50.92)	153.42 (53.64)	0.017
Cadence-fast (number/min)	119.2 (35.68)	119.92 (35.57)	0.951	119.25 (23.41)	120.52 (24.82)	0.163
Step time *L*-fast (sec)	0.60 (0.35)	0.57 (0.27)	0.327	0.53 (0.19)	0.53 (0.17)	0.254
Step time *P*-fast (sec)	0.61 (0.43)	0.60 (0.42)	0.819	0.52 (0.15)	0.52 (0.15)	0.410
Step length *L*-fast (cm)	71.08 (15.23)	68.93 (17.38)	0.378	68.33 (16.01)	74.07 (15.15)	0.007
Step length *P*-fast (cm)	68.33 (16.01)	68.38 (17.74)	0.951	72.06 (16.37)	74.23 (15.18)	0.023
Single leg stance *L*-fast (%)	33.6 (7.73)	34.63 (7.0)	0.083	35.17 (4.1)	35.22 (4.13)	0.932
Single leg stance *P*-fast (%)	34.87 (7.52)	34.93 (7.62)	0.343	33.51 (5.95)	35.4 (3.98)	0.028

**Table 3:** Comparing results of people with different disability level (EDSS).

Parameter	EDSS (1.5–4) *n* = 12	EDSS (4.5–7) *N* = 11	Mann–Whitney test*p*
	mean difference (SD)	mean difference(SD)	
Berg balance scale (0–56)	1.00 (1.27)	2.81 (2.35)	0.041
Mini-BESTest (0–28)	1.33 (1.30)	0.90 (0.70)	0.517
TUG (sec)	−0.02 (0.67)	−1.28 (3.91)	0.538
TUG-with dual cognitive task (sec)	−0.64 (1.58)	−2.77 (6.46)	0.806
ABC	−0.63 (11.52)	−8.17 (16.26)	0.121
FESI-I	0.00 (2.73)	2.00 (6.91)	0.278
MSWS-12	−2.83 (3.43)	0.27 (5.9)	0.138
Velocity-normal (cm/sec)	11.00 (14.38)	−0.70 (8.48)	0.036
Cadence-normal (number/min)	4.50 (5.66)	1.45 (5.01)	0.176
Step time *L*-normal (sec)	−0.02 (0.05)	−0.02 (0.05)	0.599
Step time *P*-normal (sec)	−0.01 (0.02)	0.07 (0.19)	0.264
Step length *L*-normal (cm)	2.85 (5.08)	4.10 (15.07)	0.242
Step length *P*-normal (cm)	3.07 (6.96)	2.39 (15.31)	0.218
Single leg stance *L*-normal (%)	0.15 (1.47)	0.42 (1.78)	0.644
Single leg stance *P*-normal (%)	2.61 (5.29)	0.30 (1.93)	0.281
Velocity-fast (cm/sec)	−0.05 (18.49)	−0.46 (10.05)	0.667
Cadence-fast (number/min)	−1.45 (8.04)	3.09 (9.25)	0.281
Step time *L*-fast (sec)	0.00 (0.02)	−0.06 (0.13)	0.059
Step time *P*-fast (sec)	0.00 (0.02)	−0.01 (0.06)	0.193
Step length *L*-fast (cm)	0.58 (6.86)	−2.34 (3.88)	0.356
Step length *P*-fast (cm)	1.52 (7.68)	−1.56 (6.86)	0.295
Single leg stance *L*-fast (%)	0.50 (2.14)	1.60 (2.61)	0.217
Single leg stance *P*-fast (%)	−0.00 (3.57)	0.13 (2.91)	0.805

**Table 4:** Follow-up effect in experimental group.

Parameter	Baseline-mean order in Friedman test	4 weeks-mean order in Friedman test	Follow-up-mean order in Friedman test	Friedman *p*
Berg balance scale (0–56)	1.41	2.50	2.09	0.001
Mini-BESTest (0–28)	1.47	2.38	2.16	0.001
TUG (sec)	2.03	2.00	1.97	0.984
TUG-cogni (sec)	2.19	2.06	1.75	0.444
T25FT (sec)	2.00	1.88	2.13	0.779
9PHT-right (sec)	2.25	1.94	1.81	0.444
9PHT-left (sec)	2.38	1.94	1.69	0.144
SDMT	1.38	2.04	2.58	0.009
ABC	2.07	1.96	1.96	0.947
FESI-I	2.09	2.03	1.88	0.782
MSWS-12	2.34	1.81	1.84	0.219
Velocity-normal (cm/sec)	1.94	2.06	2.00	0.946
Cadence-normal (number/min)	1.83	2.28	1.89	0.348
Step time *L*-normal (sec)	2.25	1.53	2.22	0.041
Step time *P*-normal (sec)	2.28	1.75	2.00	0.240
Step length *L*-normal (cm)	1.78	2.22	2.00	0.411
Step length *P*-normal (cm)	1.83	2.17	2.00	0.607
Single leg stance *L*-normal (%)	1.83	2.06	2.11	0.607
Single leg stance *P*-normal (%)	1.83	2.17	2.00	0.607
Velocity-fast (cm/sec)	2.03	2.19	1.78	0.448
Cadence-fast (number/min)	2.06	2.25	1.69	0.234
Step time *L*-fast (sec)	2.00	1.78	2.22	0.385
Step time *P*-fast (sec)	2.03	1.86	2.11	0.720
Step length *L*-fast (cm)	2.06	2.00	1.94	0.946
Step length *P*-fast (cm)	2.11	2.33	1.56	0.056
Single leg stance *L*-fast (%)	1.83	2.11	2.06	0.678
Single leg stance *P*-fast (%)	2.33	2.11	1.56	0.056

## Data Availability

The data used to support the findings of this study are included within the article.
